# Uncovering Alzheimer's Disease Related Dementias Patterns using Machine Learning in a LMIC Setting

**DOI:** 10.1002/alz70856_105467

**Published:** 2026-01-07

**Authors:** Jasmit Shah, Samuel Gitau, Sheila Waa, Udunna Anazodo, James H. Cole, Karen Blackmon, Thomas Thesen, Chinedu Udeh‐Momoh

**Affiliations:** ^1^ Brain and Mind Institute, Aga Khan University, Nairobi, Kenya; ^2^ Aga Khan University, Nairobi, Kenya; ^3^ McGill University, Montreal, QC, Canada; ^4^ Medical Artificial Intelligence (MAI) Laboratory, Crestview Radiology Limited, Lagos, Nigeria; ^5^ University College London (UCL), London, United Kingdom; ^6^ Global Brain Health Institute, University of California, San Francisco, NC, USA; ^7^ Wake Forest University, School of Medicine, Winston‐Salem, NC, USA

## Abstract

**Background:**

Neurodegenerative disease prevalence is projected to triple in the next 30 years, with sub‐Saharan Africa facing an acute brain health crisis due to population aging and a high vascular risk burden. East Africa, where 80% of the population is under 35, is expected to experience rapid brain aging and the world's highest neurological disease burden in terms of disability‐adjusted life‐years. Despite this, research on brain aging specific to this population is limited. Machine learning (ML) models trained on magnetic resonance imaging (MRI) scans offer promising diagnostic and prognostic insights for early detection of Alzheimer's disease and related dementias (ADRD). This study aims to develop an ensemble ML model tailored to improve early diagnosis.

**Methods:**

We will curate a neuroimaging database (Kenya‐NeuroBank) consisting of T1‐weighted MRI scans, demographic, and clinical data from ADRD diagnostic services at the Aga Khan University Hospital, Nairobi. This dataset, encompassing neurotypical and ADRD‐affected brains, will be used to train an ensemble ML model for ADRD prediction in the Kenyan population. Additionally, we will identify key brain structures most associated with ADRD using ML‐based feature selection techniques. Model validation will be conducted using complementary clinical datasets from Kenya and available datasets from the African American cohort (such as Wake Forest Alzheimer's Disease Research Center data and HABS‐HD study data), enabling comparisons across populations.

**Results:**

Currently, as we curate the Kenya‐NeuroBank database, 136 participant data is available. Of these 67.6% (*n* = 92) are females and the mean age is 51.7 years (SD=11.4). We anticipate creating the database to around 1000 participants. We expect our ML model to accurately classify ADRD cases, identify population‐specific structural biomarkers, and improve early detection of neurodegenerative risks in East Africa. Comparative validation with Western datasets will highlight unique environmental and lifestyle influences on ADRD pathology.

**Conclusion:**

This study addresses a critical gap in neuroimaging research in sub‐Saharan Africa, providing a tailored ML approach for ADRD prediction. By improving early diagnosis and identifying population‐specific biomarkers, our findings will contribute to personalized healthcare strategies, earlier interventions, and better patient outcomes. This work also has broader implications for brain health research across Africa.

